# RETRACTION: Effect of Two Different Peritoneal Dialysis Modalities on Wound Infection in Peritoneal Dialysis Patients: A Meta‐Analysis

**DOI:** 10.1111/iwj.70411

**Published:** 2025-03-21

## Abstract

**RETRACTION**: TangL. , ChengQ. , QingY. , YuJ. , YanX. , and LiaoC. , “Effect of Two Different Peritoneal Dialysis Modalities on Wound Infection in Peritoneal Dialysis Patients: A Meta‐Analysis,” International Wound Journal 21, no. 4 (2024): e14800, 10.1111/iwj.14800.38546145 PMC10976805

The above article, published online on 28 March 2024, in Wiley Online Library (http://onlinelibrary.wiley.com/), has been retracted by agreement between the journal Editor in Chief, Professor Keith Harding; and John Wiley & Sons Ltd. Following an investigation by the publisher, all parties have concluded that this article was accepted solely on the basis of a compromised peer review process. The editors have therefore decided to retract the article. The authors did not respond to our notice regarding the retraction.

**RETRACTION**: L. Tang , Q. Cheng , Y. Qing , J. Yu , X. Yan , and C. Liao , “Effect of Two Different Peritoneal Dialysis Modalities on Wound Infection in Peritoneal Dialysis Patients: A Meta‐Analysis,” International Wound Journal 21, no. 4 (2024): e14800, 10.1111/iwj.14800.38546145 PMC10976805

The above article, published online on 28 March 2024, in Wiley Online Library (http://onlinelibrary.wiley.com/), has been retracted by agreement between the journal Editor in Chief, Professor Keith Harding; and John Wiley & Sons Ltd. Following an investigation by the publisher, all parties have concluded that this article was accepted solely on the basis of a compromised peer review process. The editors have therefore decided to retract the article. The authors did not respond to our notice regarding the retraction.

